# Structural Insights into the Inhibition of Cytosolic 5′-Nucleotidase II (cN-II) by Ribonucleoside 5′-Monophosphate Analogues

**DOI:** 10.1371/journal.pcbi.1002295

**Published:** 2011-12-08

**Authors:** Franck Gallier, Perrine Lallemand, Maïa Meurillon, Lars P. Jordheim, Charles Dumontet, Christian Périgaud, Corinne Lionne, Suzanne Peyrottes, Laurent Chaloin

**Affiliations:** 1 Institut des Biomolécules Max Mousseron (IBMM), UMR 5247, CNRS – Universités Montpellier 1 et 2, Université Montpellier 2, Montpellier, France; 2 Centre d'études d'agents Pathogènes et Biotechnologies pour la Santé (CPBS), UMR 5236, CNRS – Universités Montpellier 1 et 2, Montpellier, France; 3 Centre de Recherche de Cancérologie de Lyon (CRCL), INSERM U1052, CNRS UMR 5286 – Université Claude Bernard Lyon 1, Lyon, France; University of Houston, United States of America

## Abstract

Cytosolic 5′-nucleotidase II (cN-II) regulates the intracellular nucleotide pools within the cell by catalyzing the dephosphorylation of 6-hydroxypurine nucleoside 5′-monophosphates. Beside this physiological function, high level of cN-II expression is correlated with abnormal patient outcome when treated with cytotoxic nucleoside analogues. To identify its specific role in the resistance phenomenon observed during cancer therapy, we screened a particular class of chemical compounds, namely ribonucleoside phosphonates to predict them as potential cN-II inhibitors. These compounds incorporate a chemically and enzymatically stable phosphorus-carbon linkage instead of a regular phosphoester bond. Amongst them, six compounds were predicted as better ligands than the natural substrate of cN-II, inosine 5′-monophosphate (IMP). The study of purine and pyrimidine containing analogues and the introduction of chemical modifications within the phosphonate chain has allowed us to define general rules governing the theoretical affinity of such ligands. The binding strength of these compounds was scrutinized *in silico* and explained by an impressive number of van der Waals contacts, highlighting the decisive role of three cN-II residues that are Phe 157, His 209 and Tyr 210. Docking predictions were confirmed by experimental measurements of the nucleotidase activity in the presence of the three best available phosphonate analogues. These compounds were shown to induce a total inhibition of the cN-II activity at 2 mM. Altogether, this study emphasizes the importance of the non-hydrolysable phosphonate bond in the design of new competitive cN-II inhibitors and the crucial hydrophobic stacking promoted by three protein residues.

## Author Summary

Nucleotidase activity is part of a biological process that allows the cell to regulate the intracellular pools of nucleotides involved in many signaling pathways. During cancer therapy with cytotoxic nucleoside analogues, the role of cN-II is unclear. Therefore, the development of specific inhibitors against this enzyme is of great interest for understanding its implication in cancer biology and drug resistance. Ribonucleoside phosphonates are of major importance because they behave as bioisosteric analogues of the natural cN-II substrates and contain a chemically and enzymatically stable phosphorus-carbon linkage. Taking the advantages of docking methods, we predicted the inhibitory potential of these compounds. Their binding strength was explained by an impressive interaction network involving mainly three residues of the enzyme (acting as hydrophobic tweezers). These new characterized inhibitors will constitute a valuable tool for elucidating the role of cN-II in cancer cells and may be used in combination with cytotoxic nucleosidic drugs in order to increase their antitumor activity. Furthermore, the strategy taking into account the hydrophobic clamp for designing new inhibitors may be applied to other nucleotidases of the HAD family as two of the three identified residues are present in the substrate binding site of cytosolic 5′-nucleotidase III and 5′-deoxynucleotidase-I.

## Introduction

Nucleotidase activity was first described in 1934 in skeletal muscle and heart by Reis and co-workers [1]. The function of this enzyme family is to regulate the intracellular pools of nucleos(t)ides by catalyzing the dephosphorylation of nucleoside monophosphates (NMP+H_2_O↔N+PO_4_^2−^). Indeed, nucleotidases contribute to maintain nucleotide pools according to the metabolic needs of the cell through a delicate regulation of kinases and nucleotidases activities [2]. Cytosolic 5′-nucleotidase II (cN-II, EC 3.1.3.5, formerly called purine 5′-nucleotidase or high K_M_ 5′-nucleotidase) belongs to the haloacid dehalogenase (HAD) super family. Among the seven human nucleotidases differing by their specificity towards substrates and cellular localizations, five are located in the cytosol, one is mitochondrial and one is extracellular and membrane bound through a glycosylphosphatidylinisotol anchor [3], [4]. All soluble 5′-nucleotidases share a similar structural fold and a common reaction mechanism, which requires the formation of a phosphoenzyme intermediate [5]. During catalysis, the first aspartate of the DMDYT sequence (motif DXDXV/T called motif I found in all members of the HAD super family) has been shown to be phosphorylated [6]. However, only cN-II and cN-III possess a phosphotransferase activity (transfer of a phosphate group from a phosphorylated nucleoside to another nucleoside). Among all these enzymes, cN-II has several unique aspects, such as a complex regulation and substrate selectivity for IMP (inosine 5′-monophosphate) and GMP (guanosine 5′-monophosphate) [7], [8]. The active form of cN-II is a homotetramer and its activity can be regulated by several allosteric ligands such as ATP, ADP, 2,3-bisphosphoglycerate, dinucleosides polyphosphate or diadenosine tetraphosphate (activators) and inorganic phosphate (inhibitor) [8], [9], [10]. Recently, a structural explanation was proposed for the allosteric regulation by an effector such as ATP, which induces a disorder-to-order transition of helix A [11].

Aside from maintaining balanced nucleoside levels in the cell, cytoplasmic 5′-nucleotidases are likely to play an important role in the activity of nucleoside analogues used as antiviral or anticancer agents [2], [12], [13], even though monophosphorylated metabolites of these drugs are not necessarily good substrates of cN-II [14]. There are several clinical evidences of a correlation between cN-II mRNA expression or activity in cancer cells and the outcome of patients treated with cytotoxic nucleoside analogues. In the case of acute myeloid leukemia, treatment involving cytarabine (ara-C) leads to a worse patient outcome when cN-II is highly expressed [15]. This effect may be explained by tissue-specificity concerns or by variants at the NT5C2 locus gene encoding for cN-II. In that case, a high number of single nucleotide polymorphisms were found to be directly associated to cN-II expression and ara-C cytotoxicity or sensitivity in AML patients receiving the related chemotherapy [16]. Another example of the implication of cN-II has been described in bone marrow cells of patients with high risk myelodysplastic syndrome. Indeed, the cN-II mRNA expression was higher for patients with ara-C-containing chemotherapies than that of healthy volunteers [17]. A correlation between clinical outcome of patients with B-cell chronic lymphocytic leukemia treated with cladribine or fludarabine and their ratio of activities between dCK and cN-II has also been reported [18]. Finally, the inhibition of cN-II expression by siRNA in astrocytoma cells was shown to induce cell death [19]. Even though there has been no study reported on the correlation between mRNA expression level and protein expression level or enzymatic activity for cN-II, these results from several independent studies concerning different malignancies and treatments strongly suggest that cN-II is directly involved in the cytotoxicity of nucleoside analogues. Thus, the modulation of cN-II could be an attractive strategy to influence the activity of cytotoxic nucleoside analogues or to directly induce cell death in cancer cells.

In order to identify the role of cN-II in cancer cells and during cancer treatment, we have initiated the search for cN-II inhibitors with the development of ribonucleoside phosphonate derivatives containing a chemically and enzymatically stable phosphonate (phosphorus-carbon) bond instead of the natural and labile phosphoester (phosphorus-oxygen) bond. The main advantage of this series of analogues resides in the presence of its P-C bond that is non-hydrolysable by cN-II and one would expect a full inhibition of the nucleotidase activity upon binding of the phosphonate analogue. Here, we focused our attention to β-modified-phosphonates in which a polar group (*i.e.* hydroxyl function) was introduced in the 5′-position as it could mimic the 5′-oxygen atom present in the natural nucleoside monophosphate. With this small subset of chemical compounds, we extensively used molecular docking to predict accurately the potential inhibitors against cN-II. During the last decade, an impressive numbers of molecular docking programs (not less than 65 in 2008 [20]) have been developed, mainly based on different stochastic methods such as Monte-Carlo, molecular dynamics or genetic algorithm for the conformational sampling of the ligand. Nowadays, the most commonly used programs in drug discovery are AutoDock, Gold, FlexX, Glide, Fred, Hammerhead, Dock, ICM or Ludi [21]. Given the large diversity of programs, it can be difficult to choose the most suitable one to our protein-ligand system. Also, a tremendous effort was achieved these last years to improve the accuracy of the ligand binding prediction by developing either new scoring functions (empirical or knowledge based) or by including water molecules or by introducing some flexibility for both the ligand and protein side chains [22], [23],[24]. We present here a structure-activity study of ribonucleoside analogues bearing modifications of the 5′,6′-phosphonate chain, based on molecular docking (Gold program) and experimental *in vitro* nucleotidase activity measurements, allowing us to highlight the structural rules governing the efficacy of such inhibitors.

## Results

### Chemical synthesis of the ribonucleoside phosphonates

**Figure 1:**
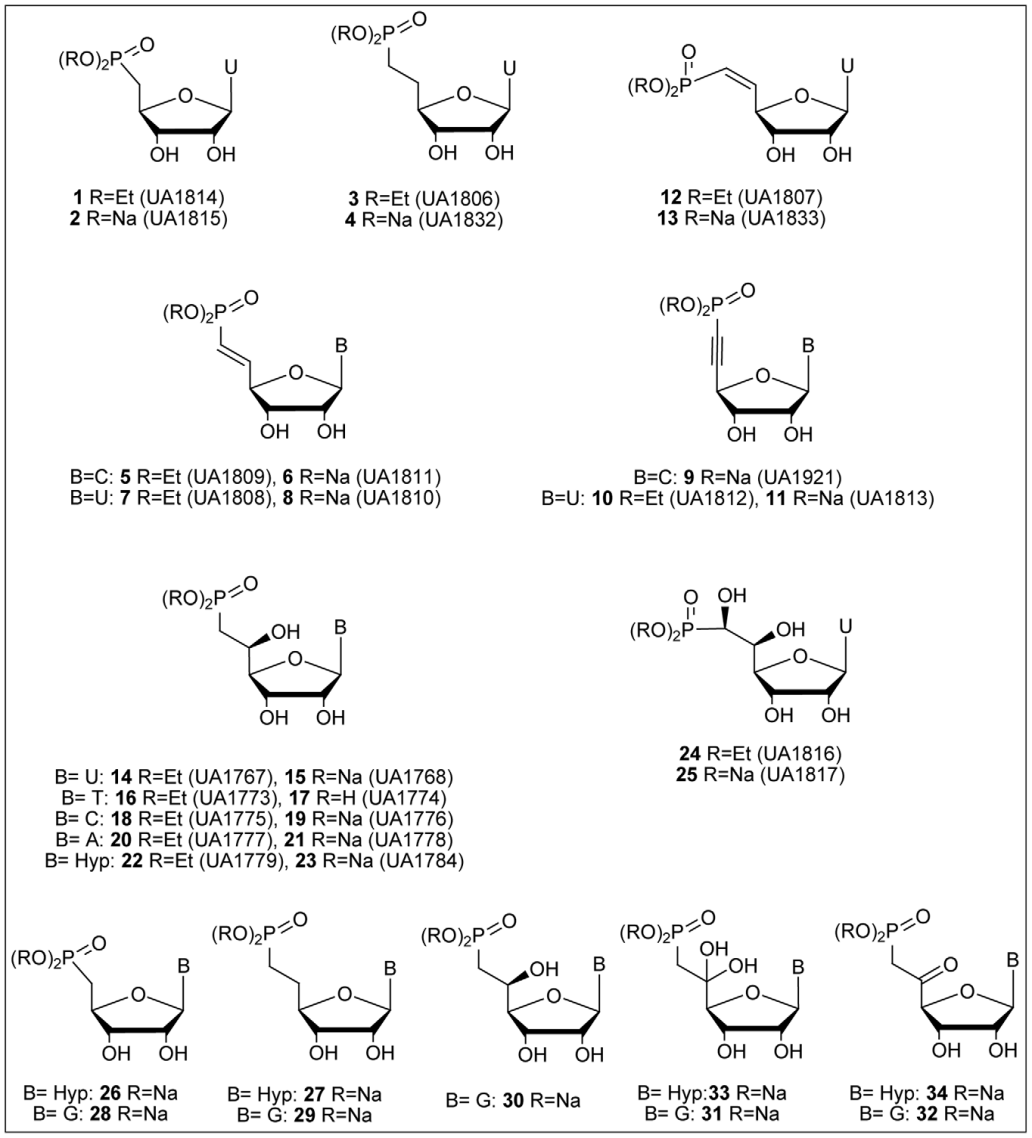
Chemical structures of the ribonucleoside phosphonate analogues evaluated for their inhibition potential against cN-II activity (compound number from 1 to 25) and designed derivatives that could not be evaluated *in vitro* (virtual compounds 26 to 32).

**Figure 2:**
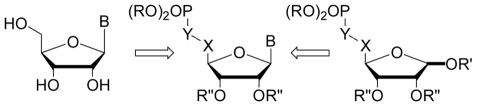
Schematic representation of the chemical retrosynthetic pathways used for the studied derivatives.

Thirty-four phosphonate analogues (incorporating a chemically and enzymatically stable P-C bond) were designed (Figure 1) as mimics of nucleoside 5′-monophosphates and potential inhibitors of cN-II. Among the 25 synthesized analogues, twelve possess a hydroxyl group in β-position related to the phosphorus atom (*i.e.* the 5′-carbon of the ribose moiety). Briefly, two synthetic routes (Figure 2) were envisaged to reach the targeted compounds: an osidic (involving the synthesis of a phosphonate-sugar intermediate and further condensation of various heterocycles) or a nucleosidic strategy. Both routes have been explored previously (see details in the materials and methods section). The phosphonate analogues were representative of most of the ribonucleoside monophosphates present in mammalian cells (AMP, CMP, UMP, TMP and IMP) even if we hypothesized that the hypoxanthine nucleobase may be preferred by cN-II. Within, the β-hydroxyphosphonate series the guanine derivative was not available due to synthetic issues [25]. As the chemistry of uridine derivatives is usually the easiest to perform, we initially obtained and tested a set of modified compounds bearing uracil as nucleobase. Nevertheless, to compensate the lack of hypoxanthine and guanine derivatives, we included these analogues (see compounds **26** to **34**) in the docking study, even if these compounds will not be immediately available for the biological assay.

In addition to the variation of the nucleobase, several modifications of the 5′,6′-phosphonate chain were studied to evaluate the influence of its rigidity or flexibility upon affinity for cN-II. Thus, a triple, a double or a single carbon-carbon bond connected to the phosphorus atom was introduced in this critical position (compounds **9–11**, **5–8**, **12**, **13** and **3–4**, respectively). Furthermore, the effect of a polar group (hydroxyl) in the 5′-position was evaluated and compared either to compound **4** (fully saturated derivative) or compound **25** in which a second hydroxyl group was added (meaning two hydroxyl groups on the 5′ and the 6′ position in respect to the phosphonate function). Four additional compounds were designed for the *in silico* screening with either a carbonyl function (**32** or **34**) or two hydroxyl groups (**31** or **33**) in the 5′ position (these two forms are in equilibrium in aqueous solution). Despite our efforts, these compounds were not obtained due to their low chemical stability in the final deprotection steps. This allows us to identify the precise role of this group and to modify the polar environment in this area that may induce changes in the hydrogen bonds network with cN-II residues.

### Docking of the ribonucleoside analogues into the active site of cN-II

In order to predict the potential of ribonucleoside phosphonates as cN-II inhibitors, molecular docking was carried out using the high resolution crystal structure of cN-II (2JC9) by targeting the active site formed by the DMDYT motif and the magnesium ion. Docking poses were classified according to their relative score (arbitrary units depending on the scoring function) that evaluates as good as possible the free binding energy by summing the different atomic interactions between the compound studied and the protein residues. It is important to note that the individual score value by itself is not meaningful and must be compared to those of the natural substrates of cN-II (IMP). Also, the docking score only gives an evaluation of the affinity of a molecule for the active site but not its ability to be catalyzed (to approach this chemical reaction step, quantum mechanics coupled to molecular dynamics has to be performed).

**Figure 3:**
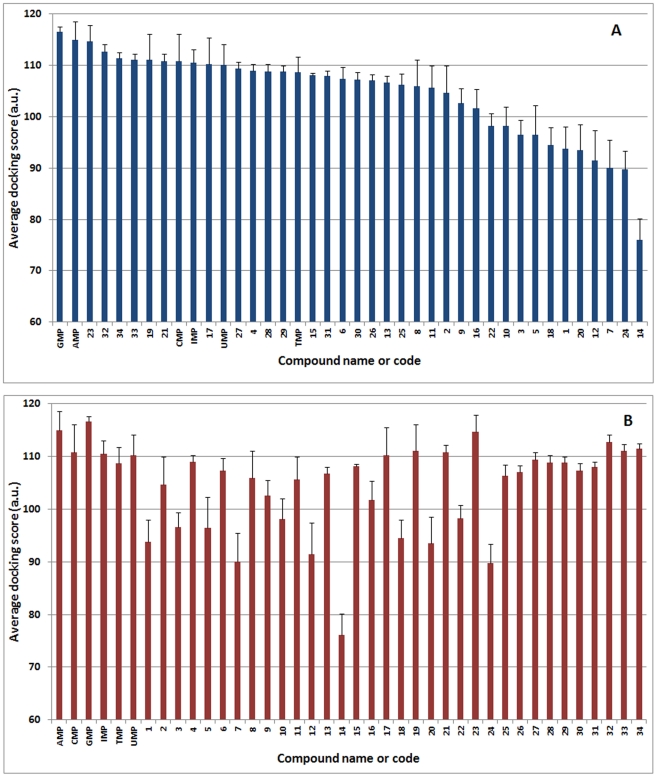
Average docking score of the studied derivatives and natural nucleoside monophosphates classified either by ranking score from the highest to the lowest (A) or by compound number (B).

**Figure 4:**
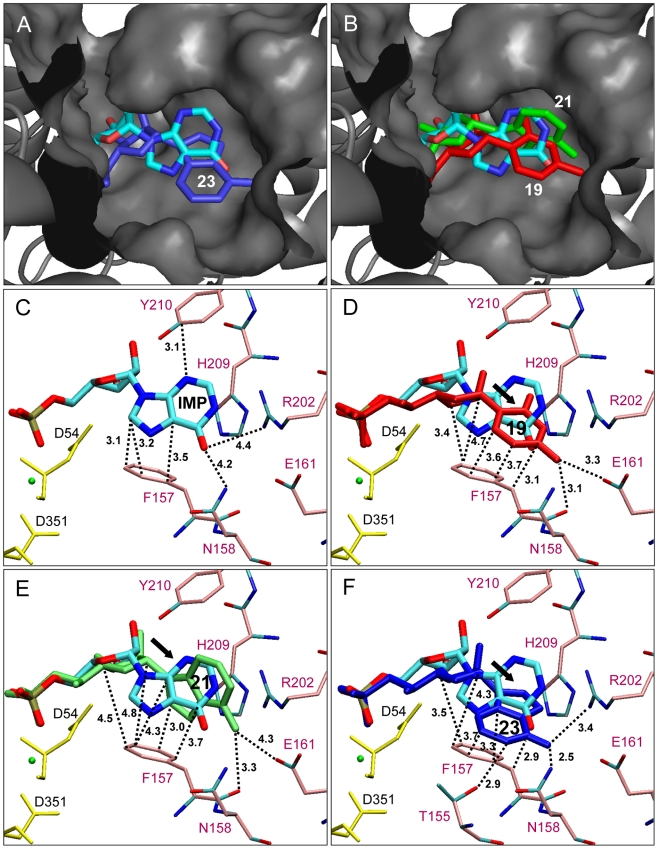
Docking results obtained with the ribonucleoside phosphonate analogues. (A) Docking pose of derivative **23** (dark blue) compared to that of **IMP** (cyan thick sticks); IMP binding site is shown by surface representation and secondary structure elements of cN-II are in grey. (B) Docking poses of compounds **19** (red) and **21** (green) compared to that of **IMP**. Atomic details of the interaction between **IMP** (C) or derivative **19** (D) or **21** (E) or **23** (F) and protein residues; magnesium ion is represented in green ball model, aspartic residues are shown in yellow sticks and other residues are shown in pink sticks model. Distances between IMP and protein residues are indicated in Å. The black arrow in panels D, E and F indicates the nucleobase/ribose displacements inducing a weakening of the binding to aromatic residues.

**Figure 5:**
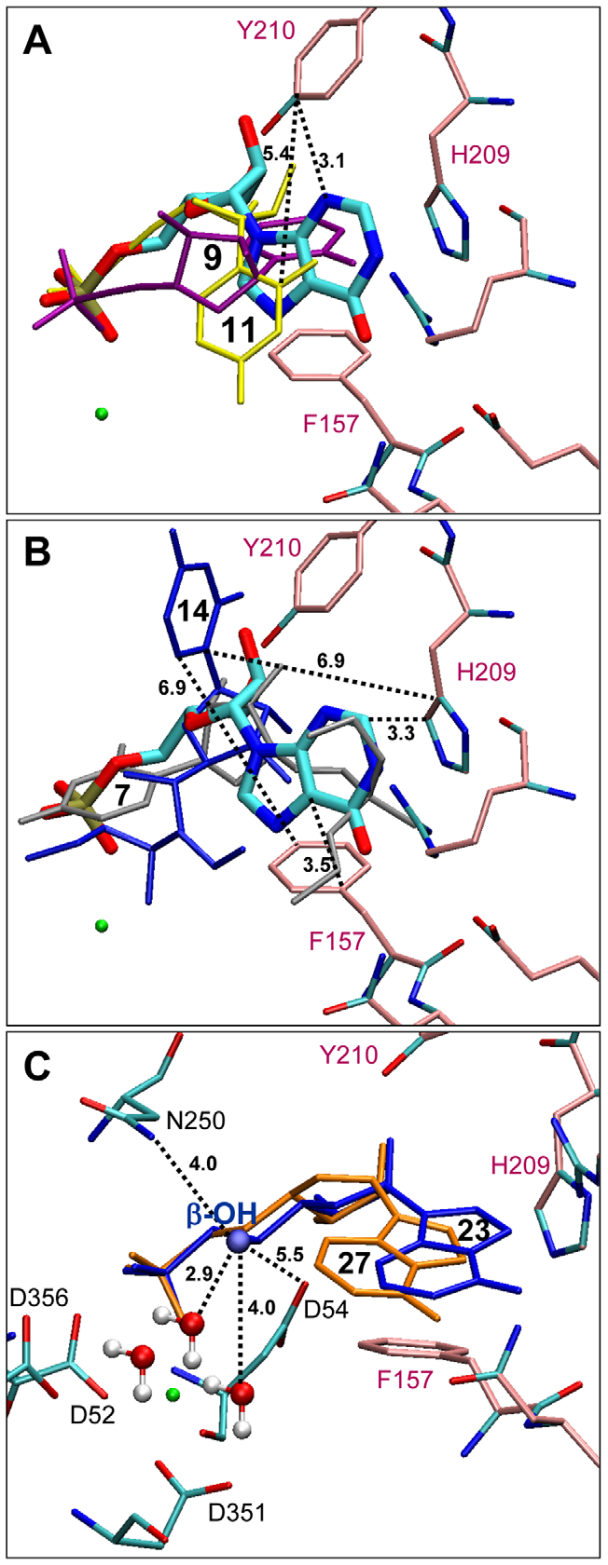
(A) Docking poses for middle score analogues, 9 (purple) and 11 (yellow) showing an increased distance between their base and Tyr 210. (B) Binding modes obtained for the lowest score compounds, **7** (grey) and **14** (dark blue); distances between the nucleobase of **14** and aromatic residues (His 209, Phe 157) are indicated in Å while compound **7** adopted a fully inverted orientation. (C) Importance of the β-hydroxyl group for the binding of the hypoxanthine analogues to cN-II: docking poses obtained with compound **27** (orange) lacking the β-hydroxyl group (ball model) and compound **23** (dark blue). Water molecules are shown in ball and stick model and distance in Å.

As shown in Figure 3, half of the compounds exhibited a docking score around 110, a score that is very close to the one of IMP (110.5). This result indicates that nucleoside phosphonate derivates represent a class of compounds that mimics efficiently the nucleoside 5′-monophosphates. Remarkably, six compounds **19**, **21**, **23** and **32–34** showed higher score than the preferred natural substrate (IMP) suggesting that these analogues may constitute powerful cN-II inhibitors or at least should bind tightly to the protein. The importance of the negatively charged oxygens was clearly identified by comparing these compounds to their phosphonate-protected analogues (the negative charges of oxygens are masked by an ethyl group) like for instance, **18**, **20** and **22**. Moreover, the worse candidate was derivative **14** for which the phosphonate function is also protected. Hence this latter was selected and used as control in addition to derivatives **7** and **24**. This confirms the importance of the interaction of the phosphonate oxygens with nucleotidase residues (especially with aspartate residues) and magnesium. Other phosphonate analogues exhibited a lower score compared to IMP suggesting that these molecules should not inhibit efficiently cN-II as their binding capacities appear very weak. In order to identify the key interactions leading to the highest docking score, the docking pose of several analogues were analyzed in details (Figure 4). Amongst the highest ranked derivatives, one can notice numerous non-bonded interactions (van der Waals contacts) between aromatic residues and derivatives **19**, **21** and **23** compared to IMP as shown in Figure 4 and Tables 1–4 (as compounds **32–34** were not available for further biological assays, we focused hereafter on these three existing analogues). In particular, Phe 157 forms a large number of contacts with the phosphonate analogue. For derivative **23**, not less than 46 contacts were observed with Phe 157 among the 76 contacts counted for this compound. A similar interaction is encountered between Phe 157 and compounds **19** or **21**, with 41 and 26 contacts, respectively. In contrast, the contacts observed between IMP and Phe 157 were less abundant (only 27) and this can be explained by a “base-ribose” shifting encountered only for the phosphonate series, bringing these compounds closer to Phe 157 (Figure 4D, 4E and 4F). Interestingly, for derivative **21**, only 26 contacts were observed with Phe 157 (comparable to IMP) but the binding is reinforced by 30 additional non-bonded contacts with His 209 and Tyr 210, leading to a high docking score (see Table S1 for the listing of atomic interactions). As soon as these three non-bonded interactions disappeared or are weakened, docking scores are significantly decreased and therefore the binding free energy is increased as shown for compound **9** (sum of the three main non-bonded interactions is 36), **14** (sum is 31), **7** (sum is 5) or **11** (sum is 0) for instance (Tables 5–8). Compounds **9** and **11** exhibited lower docking score than **19** or **21** due to positioning of the nucleobase that is not deeply inserted in the active site (Figure 5A) and led to a reduction of non-bonded contacts (distances between the nucleobase and Tyr 210 are larger). However, the coulombic interactions between the phosphonate oxygens and aspartic residues (with the magnesium connection) are preserved. This is not the case for the lowest docking-score compounds as seen for **7** and **14**, where the oxygens of the phosphonate are still protected and consequently these coulombic interactions are missing (Figure 5B). The masking of the charges of the phosphonate functionality is so detrimental that compound **7** was found to bind cN-II on the other way round with its nucleobase moiety in place of the phosphonate chain. This explains why the number of hydrogen bonds and non-bonded contacts was dramatically reduced. Phe 157 constitutes one of the major residues involved in the binding of cN-II ligands and acts in synergy with His 209 and Tyr 210 to form a hydrophobic clamp trapping the nucleobase and the ribose moiety. As shown in Figure 5 (panels A and B), the distances between the nucleobase and the aromatic residues were increased for lower score compounds compared to those of IMP (from 3.1 to 5.4 Å with Tyr 210 and from 3.5 to 6.9 Å with Phe 157 and 3.3 to 6.9 Å with His 209). Furthermore, the electrostatic interactions occurring between aspartate residues (52 and 54 and 356) and phosphonate oxygens were essential as seen by the loss of the binding potential and the activity of all phosphonate analogues bearing protected oxygen atoms. In parallel, these oxygens are bound to the Lys 292 side chain through strong ionic interactions. Lys 292 is a highly conserved residue in all nucleotidase of the HAD family that contributes directly in the proposed nucleotidase reaction mechanism [26].

**Table 1:** Atomic interactions measured between cN-II residues and **IMP** (natural substrate).

Interactions (IMP)		H-bonds		Non-bonded
		S-L	M-L	
IMP	MET 53	0	1	0
IMP	ASP 54	0	2	7
**IMP**	**PHE 157**	**0**	**0**	**27**
IMP	VAL 205	0	0	2
IMP	ASP 206	0	0	1
**IMP**	**HIS 209**	**1**	**0**	**9**
**IMP**	**TYR 210**	**3**	**0**	**9**
IMP	LYS 215	0	0	1
IMP	THR 249	1	0	0
IMP	ASN 250	0	0	3
IMP	SER 251	0	0	5
IMP	TYR 255	0	0	1
IMP	LYS 292	1	0	0
**Sums**		**6**	**3**	**65**

Non-bonded contacts included all carbon-carbon interactions shorter than 4.8 Å are listed. Hydrogen bonds (using a cutoff of 3.5 Å) are separated in two groups S-L (Side chain-ligand) and M-L (Main chain or backbone-Ligand). The residues forming the hydrophobic pocket that are discussed in the text are denoted in bold.

**Table 2 pcbi-1002295-t002:** Atomic interactions measured between cN-II residues and derivative **19** (one of the three best ranked compounds).

Interactions (19)		H-bonds		Non-bonded
		S-L	M-L	
19	MET 53	0	1	0
19	ASP 54	0	1	5
**19**	**PHE 157**	**0**	**1**	**41**
19	ASN 158*	0	0	1
19	GLU 161*	1	0	0
19	VAL 205	0	0	2
**19**	**HIS 209**	**0**	**0**	**7**
**19**	**TYR 210**	**1**	**0**	**3**
19	THR 249	1	0	0
19	ASN 250	1	1	6
19	SER 251	0	0	2
19	LYS 292	1	0	0
**Sums**		**5**	**4**	**67**

For details, see legend of Table 1. The star indicates residues corresponding to the contact differences observed between IMP and **19**.

**Table 3 pcbi-1002295-t003:** Atomic interactions measured between cN-II residues and derivative **21** (one of the three best ranked compounds).

Interactions (21)		H-bonds		Non-bonded
		S-L	M-L	
21	MET 53	0	1	0
21	ASP 54	0	2	6
**21**	**PHE 157**	**0**	**1**	**26**
21	VAL 205	0	0	6
21	ASP 206	1	0	3
**21**	**HIS 209**	**0**	**0**	**20**
**21**	**TYR 210**	**3**	**0**	**10**
21	LYS 215	1	0	1
21	THR 249	1	0	1
21	ASN 250	0	0	7
21	SER 251	0	0	6
21	LYS 292	1	0	0
**Sums**		**7**	**4**	**86**

For details, see legend of Table 1.

**Table 4:** Atomic interactions measured between cN-II residues and derivative **23** (one of the three best ranked compounds).

Interactions (23)		H-bonds		Non-bonded
		S-L	M-L	
23	MET 53	0	1	0
23	ASP 54	0	2	6
23	THR 155*	0	0	2
**23**	**PHE 157**	**0**	**0**	**46**
23	ASN 158*	0	0	2
23	VAL 205	0	0	1
**23**	**HIS 209**	**0**	**0**	**7**
**23**	**TYR 210**	**1**	**0**	**4**
23	LYS 215	1	0	0
23	THR 249	1	0	0
23	ASN 250	0	0	6
23	SER 251	0	0	2
23	LYS 292	1	0	0
**Sums**		**4**	**3**	**76**

For details, see legend of Table 1. The star indicates residues corresponding to the contact differences observed between IMP and **23**.

**Table 5:** Atomic interactions measured between cN-II residues and the middle ranked compound **9**.

Interactions (9)		H-bonds		Non-bonded
		S-L	M-L	
9	ASP 54	1	0	5
**9**	**PHE 157**	**0**	**0**	**28**
**9**	**HIS 209**	**0**	**0**	**6**
**9**	**TYR 210**	**0**	**0**	**2**
9	THR 249	1	0	0
9	ASN 250	1	1	8
9	LYS 292	1	0	0
**Sums**		**4**	**1**	**49**

For details, see legend of Table 1.

**Table 6 pcbi-1002295-t006:** Atomic interactions measured between cN-II residues and the middle ranked compound **11**.

Interactions (11)		H-bonds		Non-bonded
		S-L	M-L	
11	ASP 54	0	0	5
11	ASN 250	0	0	6
11	SER 251	0	0	4
**Sums**		**0**	**0**	**15**

For details, see legend of Table 1.

**Table 7 pcbi-1002295-t007:** Atomic interactions measured between cN-II residues and the low ranked derivative **7**.

Interactions (7)		H-bonds		Non-bonded
		S-L	M-L	
**7**	**PHE 157**	**0**	**0**	**5**
7	ASN 158	0	0	1
**Sums**		**0**	**0**	**6**

For details, see legend of Table 1.

**Table 8:** Atomic interactions measured between cN-II residues and the low ranked derivative **14**.

Interactions (14)		H-bonds		Non-bonded
		S-L	M-L	
14	ASP 54	0	0	4
14	THR 155	0	0	2
**14**	**PHE 157**	**0**	**0**	**17**
**14**	**HIS 209**	**0**	**0**	**1**
**14**	**TYR 210**	**1**	**0**	**13**
14	LYS 215	1	0	1
14	ASN 250	1	0	13
14	SER 251	0	0	10
14	LYS 292	0	0	5
14	HIS 352	1	0	6
14	GLY 355	0	0	2
14	ASP 356	0	0	1
**Sums**		**4**	**0**	**75**

For details, see legend of Table 1.

### Dissecting the binding strength of the analogues versus natural substrate

All the ribonucleoside phosphonate analogues can be decomposed in three parts: the base, the ribose and the phosphonate chain. When comparing the different nucleobases within the same chemical structure of analogues (including natural nucleotides), purines or pyrimidines exhibited a similar binding mode. This indicates that the ring size did not constitute a crucial factor. It should be highlighted that the amino group in the C6-position (adenine) or C4-position (cytosine) is advantageous because it allowed the formation of two stabilizing bonds with Phe 157 (backbone oxygen) and Glu 161 that were not possible with the carbonyl group (or oxo-function) of IMP. As shown in Figure 4 for compounds **19** and **21**, the distance between the amino group and Phe 157 was measured to 3.1 and 3.3 Å, respectively while this interaction cannot occur with IMP and is replaced by a hydrogen bond with Asn 158 (4.2 Å). The amino group is also stabilized by a hydrogen bond with Glu 161 (3.3 and 4.3 Å, for compounds **19** and **21**, respectively). Asn 158 is also involved in the interaction with **19** and **23** by forming one or two non-bonded contacts (Tables 2 and 4). This feature clearly explains why the docking score of AMP and CMP were higher than the one of IMP. Comparing hypoxanthine derivatives and IMP, analogue **23** was deeper inserted in the binding site bringing its nucleobase in close proximity to aromatic residues and also Asn 158 and Arg 202. Regarding the phosphonate chain, the presence of a β-hydroxyl group was found to play an important role in the hydrogen bond network with Asp 54, Asn 250 and two water molecules (Figure 5C). These interactions are likely to influence the enzyme-catalyzed reaction rate either by stabilizing the phosphoenzyme intermediate or by helping the water molecules function. When focusing on hypoxanthine analogues, the docking scores were sensitive enough to distinguish between a saturated phosphonate and a β-hydroxyphosphonate as observed for compounds **23** and **27** (the difference between these two molecules is the presence of the β-hydroxyl group and their docking scores were 115 and 109, respectively). Indeed, the β-hydroxyl group is responsible for a significant improvement of the inhibition of the nucleotidase activity (see below). This result was further confirmed by docking of di-hydroxyl or β-keto-phosphonate derivatives (in aqueous media these two forms should be in equilibrium) such as compounds **33** and **34** for instance. Indeed, very high score were obtained suggesting a strong binding of these compounds to the enzyme (with two direct interactions with Asn 250 and water molecules). Thus, the presence of this oxygen may mimic the missing oxygen of the phosphoester bond and may increase the binding affinity. The length of the carbon chain between the phosphorus atom and the sugar ring did not appear as a decisive parameter (demonstrated by the docking score of compounds **2** and **4**) as well as the rigidity of this chain when introducing a double or triple bond (see compounds **8**, **11** and **13** for instance to be compared with **4**). Indeed, limiting the free rotation around carbon-carbon bonds in this area induced a slight reduction of the docking score. It has to be highlighted that for β-hydroxyphosphonates, the flexibility may be required for a correct positioning of the β-hydroxyl group since the docking score (and the inhibition force) of compound **15** is much higher than the previously mentioned analogues bearing a double or triple bond. The addition of a second hydroxyl group in the 6′-position was not predicted as an improvement of the binding and of the inhibitory activity (10–15% at 2 mM) of the corresponding analogue (comparing **15** and **25**).

### Nucleotidase activity is inhibited by ribonucleoside phosphonate analogues

**Figure 6:**
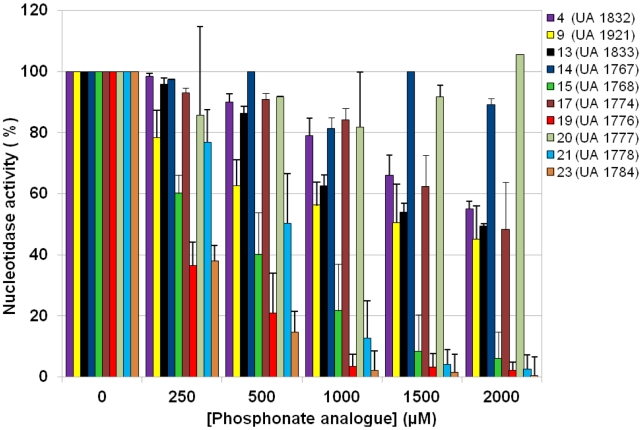
Inhibition of the nucleotidase activity by the studied phosphonate analogues. IMP was used as substrate and the cN-II activity was measured according to the inorganic phosphate release.

**Figure 7:**
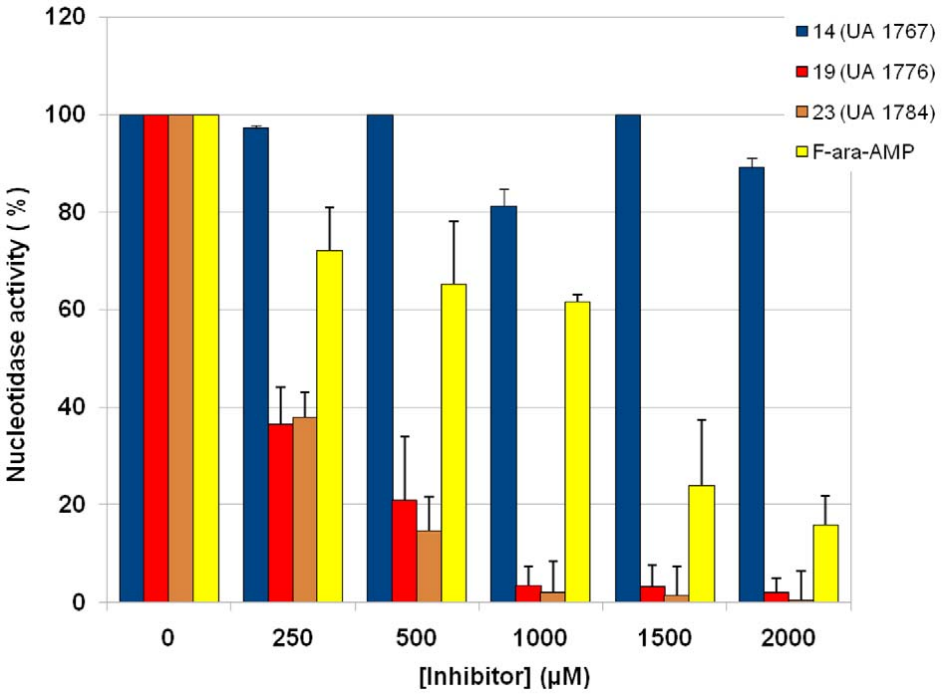
Inhibition of the nucleotidase activity by different types of cN-II inhibitors: β-hydroxyphosphonate analogues 14, 19, 23 and fludarabine monophosphate (F-ara-AMP). Compound 14 was used as a negative control.

As docking represents only a theoretical approach, all the existing phosphonate analogues (derivatives **1–25**) were challenged against cN-II in presence of its natural substrate (IMP) in order to evaluate experimentally their inhibition potential of cN-II activity. The most powerful inhibitors were derivatives **19** and **23**, able to induce a total inhibition of the nucleotidase activity at 1 mM (Figure 6). Similarly, compounds **15** and **21** were found to inhibit the enzyme activity with a faintly lower efficiency than that of compound **19** but promoting around 97% of inhibition at a concentration of 2 mM. The nucleotidase activity was partially reduced in presence of several analogues like **9** or **13** at 2 mM while no inhibition was observed for **14** and **20**. These results unravel the importance of the negatively charged oxygens of the phosphonate group. It is important to note that the nature of the nucleobase constitutes a crucial factor to promote a powerful inhibition as observed when comparing compounds **15** (uracil), **17** (thymine), **19** (cytosine), **21** (adenine) or **23** (hypoxanthine). However, even if CMP is predicted as a good ligand, it is not a privileged substrate of cN-II (very low nucleotidase activity measured in presence of CMP instead of IMP, see Figure S1) and even less an inhibitor (no change of the nucleotidase activity observed in presence of 1 mM of CMP, data not shown). Examining the effect of a double bond within the phosphonate chain, the inhibition observed for **13** was comparable to that of derivative **4** bearing a single carbon-carbon bond (Figure 6) but was found much lower than **15** for the same series (uracil with a β-hydroxyl group). This was not precisely anticipated from the docking scores (slight decrease) suggesting that the effect of the rigidity of the phosphate chain could not be really significant. However, the nucleobase-ribose displacement observed in the docking pose indicated a weaker interaction with Phe 157 that corroborates well with the inhibition results. In order to compare the inhibition efficiency of such phosphonate analogues with other inhibitors, the nucleotidase activity was measured in presence of the only available and validated cN-II inhibitor, namely fludarabine monophosphate (F-ara-AMP) (Figure 7). This AMP analogue was previously characterized [27] as a substrate of cN-II and interfered with the hydrolysis of IMP. Here, F-ara-AMP was first evaluated as a substrate in order to define the real contribution of inorganic phosphate released within our experimental assay. F-ara-AMP was found to be a weak substrate of cN-II compared to IMP (20% of F-ara-AMP hydrolyzed versus 100% with IMP, see Figure S1 and this ratio was constant at concentrations above 200 µM and up to 2 mM) but in contrast, it was able to induce a strong inhibition of the cN-II activity at high concentrations (80% at 2 mM). In conclusion, the phosphonate analogues were able to induce a stronger inhibition of the nucleotidase activity than F-ara-AMP confirming that modification of the phosphate chain was a suitable strategy for the design of new cN-II inhibitors.

### Structure-activity relationships: key features

The presence of a chemically and enzymatically stable phosphonate group, in replacement of the regular phosphoester bond was confirmed to be significantly advantageous for the inhibition of cN-II activity. The presence of a β-hydroxyl group in the phosphonate chain was considered as a major improvement whereas two hydroxyl groups were not helpful, for example, the inhibition rank for compound **25** was not higher than **4**. The presence of a carbonyl group instead of one hydroxyl at the 5′ position further confirmed this feature (compounds **32** and **34**). The length of the phosphonate chain must be correctly defined to ensure a correct positioning of the oxygen atoms in the active site allowing the interactions with Asp 54 and two water molecules present in this site. In contrast, the rigidity of this chain was found to be a disadvantage because of the imperfect positioning of the nucleobase and ribose moieties. Interestingly, compound **19** (cytosine) was found to be as potent inhibitor of the nucleotidase activity as **23** (hypoxanthine) unraveling the importance of the amino-group in the C6-position of the nucleobase. As cytosine is not a favored substrate of cN-II in terms of specificity (pyrimidine-containing nucleotides are dephosphorylated preferentially by cN-III), it was surprising that compound **19** was highly active. The docking score of CMP was also found prominent. Therefore, CMP was assayed as substrate (instead of IMP) in our experimental assay in order to control its possible hydrolysis by cN-II. As expected, a very low production of inorganic phosphate confirmed the high specificity of cN-II for purine base (Figure S1). In contrast to pyrimidine-based analogues, the docking score of F-ara-AMP (adenine derivative) was in the order of 115, which indicated a potential strong cN-II inhibitor. Within the nucleotidase structure, the substrate selectivity is governed by few residues forming the hydrophobic clamp: Phe 157, His 209 and Tyr 210. The binding is then consolidated by the unspecific electrostatic binding of phosphonate or phosphate oxygens using the magnesium ion for bridging repulsive negative charges. Finally, to reach the reactive state leading to the hydrolysis of the substrate, other residues may play an important role such as Lys 292, Thr 249 or Asp52 which must be in close proximity of the phosphate group.

## Discussion

A new family of nucleoside 5′-monophosphate analogues bearing a phosphonate bond was assayed against cN-II and some of them were shown to inhibit its nucleotidase activity. At high concentration (1.5–2 mM) a complete inhibition was observed in the case of derivatives **19** and **23**. As cN-II is also called High-K_M_-nucleotidase, this concentration range is not surprising taking into account that intracellular concentrations of NMPs are in the millimolar range (depending on the cell type and the intracellular compartment) [28], [29], [30]. The cytosine containing analogue (**19**) was found to be equivalent in terms of inhibition to the hypoxanthine and adenine counterparts. This result corroborates well the docking prediction but it was not the case for CMP (predicted as good ligand) which finally turns to be a very bad inhibitor of cN-II *in vitro*. This confirms that cN-II is highly specific for purine-based nucleotides and that its nucleotidase activity does not overlap with cN-III. On the other hand, it underlines the limitation of the docking study that can be very instructive for estimating the binding affinity of a ligand but may not be considered as a valuable tool for the prediction of the entire enzyme-catalyzed reaction. For instance, the affinity of cN-II for CMP is predicted as good as for IMP by docking but it does not indicate that they are equivalent as substrates (as shown *in vitro*). Therefore, a strong binding is not sufficient for predicting a high catalytic efficiency and the dynamics of the protein and/or the residential time of the substrate in the active site are likely to play a complementary role for an efficient catalysis. In any case, the docking is accurate for predicting competitive inhibitors, at least in part because the enzyme reaction cannot occur. Interestingly, this enzyme exhibited high ligand versatility as it accommodated the binding of hypoxanthine, adenine, guanine and cytosine derived analogues. Indeed, the presence of both types of residues (positively and negatively charged) deeply buried in the binding site may explain how the enzyme affords the interaction with either an oxo group or an amino group (residues Glu 161 and Arg 202 in addition to Asn 158). The inhibition mediated by the analogue **19** was supported by the impressive number of van der Waals interactions between the nucleobase and Phe 157. For the compound **21** (adenine), the strong binding is due to additional contacts with His 209 and Tyr 210 (still in addition to those with Phe 157). The most important structural features governing a tight interaction (and consequently a powerful inhibition) were first the negatively charged phosphonate group, secondly, the addition of a β-hydroxyl group and finally, the presence of an amino group in position 4 or 6 for pyrimidine and purine base, respectively. The three water molecules (already present in the crystal and conserved for docking) play an important role in bridging the β-hydroxyl group to the aspartate residues and the magnesium ion. The large contribution of hydrophobic contacts constitutes the major basis for predicting a good inhibitor by examining the interaction between the ribonucleoside moiety and Phe 157, His 209 or Tyr 210. Indeed, these aromatic residues act as hydrophobic tweezers trapping the ribonucleoside through stacking interactions. The energetic contribution of an aromatic interaction (π-π interactions that are caused by intermolecular overlapping of p-orbitals in π-conjugated systems) is usually estimated to about 0.5–3 kJ/mol while hydrogen bonds are much higher (5–30 kJ/mol). However, when the number of these bonds increases, this energetic contribution must be taken into account for the calculation or prediction of the free binding energy. Very recently, the crystal structure of IMP bound cN-II was solved by Wallden and co-workers [11] who introduced the D52N mutation in order to prevent IMP hydrolysis. Interestingly in this work, the role of Phe 157 was already underlined for being important in substrates recognition and selectivity. To confirm the importance of Phe 157, another docking was carried out using the F157A mutant (*in silico*) and the docking score of both natural nucleotides and phosphonates analogues were reduced by about 15 points (data not shown). Additionally, UMP/GMP bound structures were also solved by this group and besides the presence of the mutation in the active site, this allows us to compare the binding mode and validate the docking poses. Undeniably, very low rmsd (less than 1.5 Å for all-atoms) were measured for these two natural nucleotides between crystal and docking poses leading to a high confidence in the docking results presented here. To conclude, our theoretical docking study brings new insight for the development of novel and potent competitive inhibitors against cN-II including new structural key components that were found to be in good agreement with experimental results and recent crystal structures.

Many virtual screening studies have been published yet leading to the discovery of newly and potent enzyme inhibitors, but only few of them have drawn up the quantitative structural key components responsible for the inhibitory activity [31], [32]. Molecular docking has been extensively used for ligands screening and is a valuable tool to characterize strong inter-atomic interactions. Here, the docking was exploited for both the screening and the quantification of the interactions (number of van der Waals contacts and hydrogen bonds). Thus, our results will be helpful for the development of new cN-II inhibitors belonging to the nucleotide analogs family since the structural rationale is now established. A main advantage of the β-hydroxyphosphonate series is that they retain their specificity for the targeted nucleotidase as the nucleobase can be chosen accordingly (hypoxanthine for cN-II). As pointed out by Mazzon and coworkers [14], overproduction of cN-II could lead to the resistance against purine analogs but not with cytosine analogues since the dephosphorylation of gemcitabine or araC could not be attributed to any of the three studied enzymes (cdN, mdN and cN-II). In this study, the authors were the first to describe cdN and mdN inhibitors containing a thymine or a uracil as nucleobase that is in perfect agreement with the substrate selectivity of these enzymes. Interestingly, one of these two inhibitors possesses one common feature with the currently studied analogues, the phosphonate linkage. More recently, the search of new inhibitors has focused on ecto-nucleotidase [33] or ectonucleoside triphosphate diphosphohydrolase [34] but nothing has been reported yet on the cytosolic human nucleotidases. Now, within the β-hydroxyphosphonate series three candidates were identified as valuable inhibitors and can be considered as promising compounds for elucidating the *in vivo* role and implication of cN-II in the resistance phenomenon observed during cancer treatments.

## Materials and Methods

### Synthesis of ribonucleoside phosphonate analogues

The uracil series of ribonucleoside phosphonate analogues (Figure 1) were synthesized as previously described [35], [36], [37] starting from uridine (Carbosynth, UK). This nucleosidic strategy has lead to derivatives **1**–**11**, **24** and **25** (see Figure 1). (E)-Vinylphosphonate and alkynylphosphonate analogues (**5**, **7** and **10**, respectively) were obtained from a common intermediate 5′-aldehyde engaged either in a Wittig or a Corey-Fuchs type reaction. In order to reach the vinylphosphonate **12** (and then **13**) with a Z-stereochemistry, the reduction of the corresponding alkynyl phosphonate was performed in presence of Lindlar Pd. Then, we considered modification of the double-bond of compound **7** using standard hydrogenation (Pd/C) and dihydroxylation conditions to obtained derivatives **3** and **24**, respectively. All members of the β-hydroxyphosphonate nucleoside series (compounds **14** to **23**) have been synthesized, with good to moderate yields, using an exchiral pool sugar based synthetic approach [25]. All compounds were characterized in accordance with their physical and chemical properties.

### Molecular modeling and docking

The modeling of all nucleotide analogues was achieved using the Biopolymer module implemented in the Insight II program suite (Accelrys Inc.). Each phosphonate derivative was built based on the corresponding natural nucleoside for which the topology is known and subjected to energy minimization under torsion constraints in order to favor the C4′-exo (or _4_E) conformation of the ribose (the most often encountered conformation of nucleotides in the active site of polymerases having a DMDY motif [38], [39]). The five torsion angles (θ_0,1,2,3,4_) in the β-D-ribose (or furanose ring) were adjusted to values of 22.92°, −37.09°, 37.09°, −22.92° and 0° respectively [40]. The potential energy of the phosphonate nucleotides was minimized with a torsion force (set to 1000 kcal/mol) using Discover and the consistent valence force field in two phases, 1000 steps of steepest descent (SD) followed by 5000 steps of conjugate gradient with a tolerance of 0.001 kcal/mol Å (dielectric constant set to 1). The crystal structure of human cN-II [41] solved at high resolution (2JC9, 1.5 Å resolution) was used for docking of the various phosphonate analogues. A few new crystal structures of cN-II were solved since we have started our docking study, our choice to use 2JC9 was based on its better resolution and the absence of mutation in the active site (D52N) that could modify the interaction with the phosphonate group. Addition of the hydrogen atoms was achieved with GOLD (Genetic Optimization for Ligand Docking, CCDC software limited) prior to the flexible docking of the nucleotide derivatives with Gold 5.0.1 program. Briefly, 50 genetic algorithms (GA) were performed with a radius of 15 Å around the magnesium atom used as target for docking. The water molecules present in the crystal structures were retained and allowed to spin around their oxygen atom. For each docked molecule, two independent docking runs (50 GA, each) were performed using Goldscore as scoring function. The different docking poses were analyzed by the clustering method (complete linkage) from the rmsd matrix of ranking solutions. For the best ranked docking poses, the potential energy was minimized in three steps using the CHARMM (Chemistry at HARvard Macromolecular Mechanics [42], [43]) program (500 steps of SD) followed by 5000 steps of a conjugate gradient with a tolerance of 0.01 kcal/mol Å without applying any constraint. The Charmm22 topology (with CMAP, an energy correction map based on quantum mechanical calculation) was used for energy minimization and the missing parameters for the phosphonate analogues were added according to the existing ones of similar groups already present in the topology (carbon-phosphorus bond). Missing partial charges were calculated using Gasteiger-Marsili empirical atomic partial charges [44], [45]. For visualizing and analysing the molecular details of the nucleotide binding to cN-II, Pymol [46] or VMD (Visual Molecular Dynamics) software was used [47]. All the hydrogen bonds and non-bonded contacts (only between hydrophobic carbon atoms) were computed using Ligplot [48] version 4.5.3 and HBplus [49] version 3.2 using cut-off values of 3.5 Å and 4.8 Å, respectively.

### Protein expression and purification

The pET24b plasmid encoding for cN-II with a 30 aminoacids deletion in N-terminal and a His tag in C-terminal [50] was kindly provided by J. Spychala and transformed into *E. coli* BL21. Cells were grown at 37°C in Luria Broth medium containing 70 µg/mL of kanamycin up to an absorbance at 600 nm of 1 followed by induction with 1 mM isopropyl-β-D-thiogalactopyranoside (IPTG) overnight at 18°C. Harvested cells were resuspended in 20 mM Tris-HCl pH 8.0, 20 mM KCl, 1 mM EGTA, 10 mM β-mercaptoethanol supplemented with one tablet of complete EDTA-free protease inhibitors (Roche Applied Science) per 50 mL. Cells were lysed with a Cell Disruptor Z Plus series (Constant System Ltd., United Kingdom). The supernatant was clarified by two centrifugations (15320 g for 20 min and 184300 g for 30 min). The 6His-tagged cN-II-ΔN was purified at 4°C in two steps using an ÄKTA-purifier fast protein liquid chromatography equipment (GE Healthcare) connected to an His-Trap FF 5 mL column (GE Healthcare) followed by a HiLoad 16/60 Superdex 75 gel filtration column (GE Healthcare). Briefly, clarified supernatants were loaded onto the nickel affinity column pre-equilibrated in 20 mM Tris-HCl pH 7.0, 400 mM NaCl, 20 mM imidazole, 10 mM β-mercaptoethanol and cN-II was eluted with a linear gradient of imidazole from 20 to 400 mM. The fractions containing cN-II were pooled and concentrated to 1 mL using ultra-filtration device (Amicon membrane, cutoff 30 kDa, Millipore). The pool was loaded onto the gel filtration column equilibrated with 50 mM imidazole pH 6.5, 10 mM MgCl_2_, 500 mM NaCl, 1 mM DTT. The fractions containing cN-II were pooled and concentrated to 3–5 mg/mL by ultra-filtration. Aliquots of 99% pure protein were stored at −80°C with 50% glycerol. The concentration of cN-II was determined using an extinction coefficient of 67200 M^−1^ cm^−1^ at 280 nm.

### Nucleotidase activity assay

Nucleotidase activity was evaluated by measuring the inorganic phosphate release upon hydrolysis of IMP by cN-II. The Malachite Green Phosphate Assay Kit (Gentaur) is based on quantification of the green complex formed between Malachite Green, molybdate and free orthophosphate. Protein concentration and volume of the reaction were adapted for 96-wells plate according to the manufacturer recommendations and free phosphate was quantified by reading the absorbance above 570 nm on a plate reader (Tecan Sunrise). Typically, cN-II was added at a final concentration of 0.1 µM in 80 µL of buffer containing 50 mM imidazole pH 6.5, 500 mM NaCl, 10 mM MgCl_2_, 1 mM DTT on ice and the reaction was started by addition of the substrate (50 µM of IMP with an equimolar amount of Mg) and incubated at 37°C. For measuring the inhibition induced by the presence of a phosphonate analogue (concentrations range from 250 µM to 2 mM), this latter was added 10 min prior addition of IMP. After 5 min at 37°C (starting from the reaction mixture prepared on ice, therefore the actual reaction time at 37°C is less than 5 min), the reaction was stopped by addition of 20 µL of Green malachite reagent on ice and signal readouts was done 15 min later. As a control, the kinetic rate constant of the reaction was measured using a quench-flow apparatus and HPLC quantification of inosine and IMP contents (Whatman Partisphere 5-SAX column and 15 mM ammonium phosphate buffer pH 5.5 as mobile phase). By this means, we have checked that the incubation time at 37°C was appropriate and that during this time, the reaction rate was constant (steady state phase). When CMP or F-ara-AMP was assayed as potential cN-II inhibitor (since these two molecules may also release a small amount of inorganic phosphate such as IMP), the inorganic phosphate was first quantified in the absence of IMP in order to evaluate the unspecific hydrolysis and considered as negligible compared to IMP (Figure S1).

## Supporting Information

Figure S1 Control of the inorganic phosphate released by cN-II when using non favored substrates such as CMP (green) or F-ara-AMP (blue) compared to that of produced by IMP hydrolysis (red). Experimental procedure is identical as the one presented in Material and Methods. Briefly, IMP was replaced by CMP or F-ara-AMP in the concentrations range between 0 and 200 µM and incubated with cN-II (0.1 µM) for 5 min at 37°C. Inorganic phosphate was then quantified using the green Malachite reagent by reading the absorbance above 570 nm.
(PDF)

Table S1 List of the non-bonded contacts (all carbon-carbon interactions shorter than 4.8 Å) calculated between cN-II residues and IMP or compound **19**, **21** or **23**. The phosphonate analogues are designed in three parts, “ADE” for adenine, “CYT” for cytosine, “HYP” for hypoxanthine and “BDR” for β-D-ribose and “PHO” for the phosphonate chain. All distances are given in angstroms.
(PDF)
