# Supplementary figures and images for: Structural Insights into the Inhibition of Cytosolic 5′-Nucleotidase II (cN-II) by Ribonucleoside 5′-Monophosphate Analogues
Source: PLoS Comput Biol. 2011 Dec 8;7(12):e1002295. doi: 10.1371/journal.pcbi.1002295 (PMC3234209; doi:10.1371/journal.pcbi.1002295)

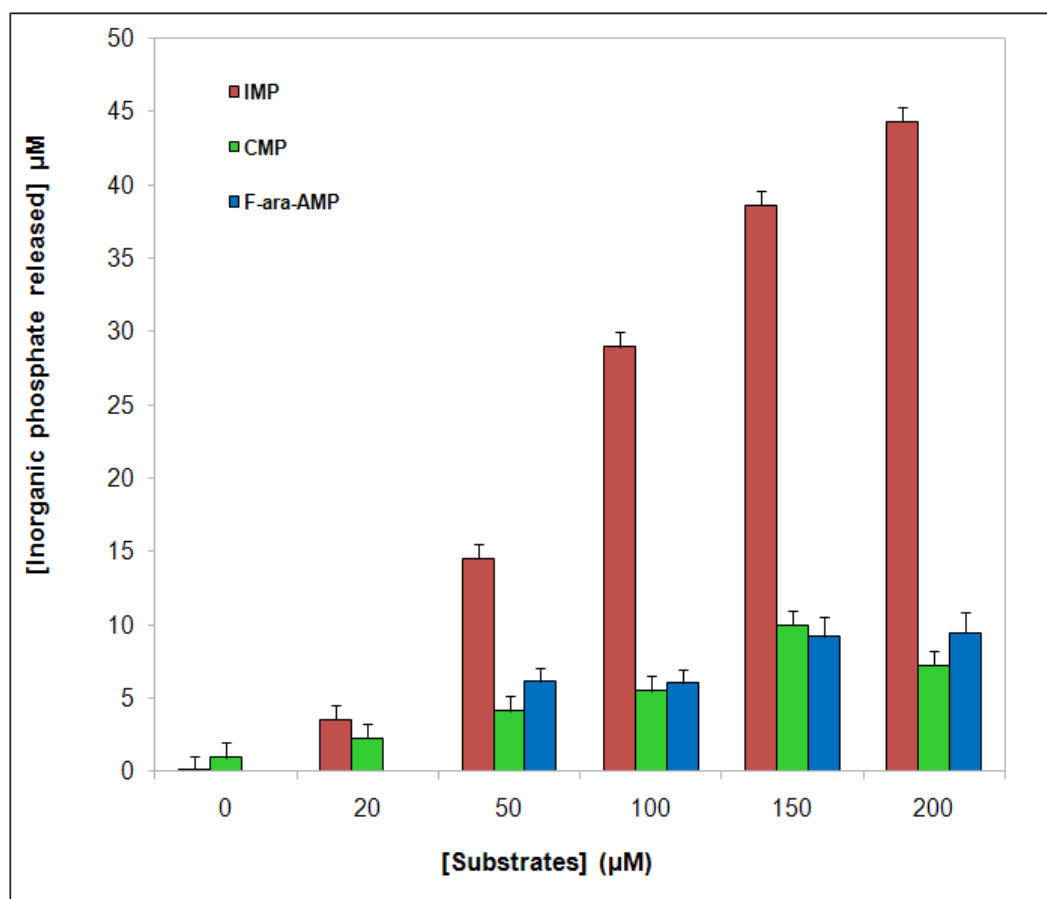

**Figure S1**

Supplement: Figure S1 — Control of the inorganic phosphate released by cN-II when using non favored substrates such as CMP (green) or F-ara-AMP (blue) compared to that of produced by IMP hydrolysis (red). Experimental procedure is identical as the one presented in Material and Methods. Briefly, IMP was replaced by CMP or F-ara-AMP in the concentrations range between 0 and 200 µM and incubated with cN-II (0.1 µM) for 5 min at 37°C. Inorganic phosphate was then quantified using the green Malachite reagent by reading the absorbance above 570 nm. (PDF) [file pcbi.1002295.s001.pdf]
