# Supplementary material for: Structural Insights into the Inhibition of Cytosolic 5′-Nucleotidase II (cN-II) by Ribonucleoside 5′-Monophosphate Analogues
Source: PLoS Comput Biol. 2011 Dec 8;7(12):e1002295. doi: 10.1371/journal.pcbi.1002295 (PMC3234209; doi:10.1371/journal.pcbi.1002295)
Supplement: Table S1 — List of the non-bonded contacts (all carbon-carbon interactions shorter than 4.8 Å) calculated between cN-II residues and IMP or compound 19, 21 or 23. The phosphonate analogues are designed in three parts, “ADE” for adenine, “CYT” for cytosine, “HYP” for hypoxanthine and “BDR” for β-D-ribose and “PHO” for the phosphonate chain. All distances are given in angstroms. (PDF) [file pcbi.1002295.s002.pdf]

IMP		Atom1	Residues		Atom2	Distance
IMP		C3'	TYR	255	CE2	4.69
IMP		C3'	SER	251	CB	4.45
IMP		C4'	SER	251	CB	3.70
IMP		C5'	SER	251	CB	4.05
IMP		C4'	SER	251	CA	3.87
IMP		C5'	SER	251	CA	4.48
IMP		C4'	ASN	250	CB	4.65
IMP		C5'	ASN	250	CB	4.70
IMP		C4'	ASN	250	C	4.41
IMP		C2'	LYS	215	CE	4.72
IMP		C4	TYR	210	CZ	4.05
IMP		C2	TYR	210	CZ	3.76
IMP		C1'	TYR	210	CZ	3.84
IMP		C2'	TYR	210	CZ	3.77
IMP		C1'	TYR	210	CE2	4.38
IMP		C2'	TYR	210	CE2	3.90
IMP		C4	TYR	210	CE1	4.40
IMP		C2	TYR	210	CE1	3.42
IMP		C2	TYR	210	CD1	4.32
IMP		C2	HIS	209	CE1	4.01
IMP		C6	HIS	209	CE1	4.17
IMP		C5	HIS	209	CE1	4.75
IMP		C4	HIS	209	CD2	4.50
IMP		C2	HIS	209	CD2	3.99
IMP		C4	HIS	209	CG	4.46
IMP		C2	HIS	209	CG	3.31
IMP		C6	HIS	209	CG	4.70
IMP		C2	HIS	209	CB	3.43
IMP		C2	ASP	206	CA	4.47
IMP		C2	VAL	205	CG1	4.76
IMP		C6	VAL	205	CG1	4.54
IMP		C5	PHE	157	CZ	4.55
IMP		C8	PHE	157	CZ	3.79
IMP		C3'	PHE	157	CZ	4.14
IMP		C5'	PHE	157	CZ	4.64
IMP		C4	PHE	157	CE2	4.64
IMP		C5	PHE	157	CE2	4.11
IMP		C8	PHE	157	CE2	3.12
IMP		C3'	PHE	157	CE2	4.57
IMP		C5'	PHE	157	CE2	4.55
IMP		C5	PHE	157	CE1	4.54
IMP		C8	PHE	157	CE1	4.43
IMP		C4	PHE	157	CD2	4.48
IMP		C6	PHE	157	CD2	4.19
IMP		C5	PHE	157	CD2	3.58
IMP		C8	PHE	157	CD2	3.23
IMP		C4	PHE	157	CD1	4.78
IMP		C6	PHE	157	CD1	4.23
IMP		C5	PHE	157	CD1	4.05
IMP		C8	PHE	157	CD1	4.50
IMP		C4	PHE	157	CG	4.56
IMP		C6	PHE	157	CG	3.72
IMP		C5	PHE	157	CG	3.55
IMP		C8	PHE	157	CG	3.98
IMP		C6	PHE	157	CB	3.44
IMP		C5	PHE	157	CB	3.80
IMP		C8	PHE	157	CB	4.78
IMP		C6	PHE	157	CA	4.80
IMP		C3'	ASP	54	CG	3.98
IMP		C4'	ASP	54	CG	4.13
IMP		C5'	ASP	54	CG	3.32
IMP		C3'	ASP	54	CB	4.59
IMP		C4'	ASP	54	CB	4.47
IMP		C5'	ASP	54	CB	3.28
IMP		C5'	ASP	54	CA	4.37

19		Atom1	Residues		Atom2	Distance
PHO		C5	SER	251	CB	4.68
PHO		C5	SER	251	CA	4.76
PHO		C5	ASN	250	CG	4.64
BDR		C5	ASN	250	CB	4.02
BDR		C4	ASN	250	CB	4.47
PHO		C5	ASN	250	CB	3.64
PHO		C5	ASN	250	C	4.36
PHO		C5	ASN	250	CA	4.22
BDR		C2	TYR	210	CZ	4.32
BDR		C1	TYR	210	CZ	4.18
BDR		C1	TYR	210	CE1	4.73
CYT		C4	HIS	209	CE1	4.48
CYT		C2	HIS	209	CE1	3.66
BDR		C2	HIS	209	CD2	4.19
CYT		C2	HIS	209	CD2	3.94
BDR		C2	HIS	209	CG	4.59
CYT		C2	HIS	209	CG	3.70
CYT		C2	HIS	209	CB	4.34
CYT		C4	VAL	205	CG1	3.99
CYT		C2	VAL	205	CG1	4.77
CYT		C5	ASN	158	CG	4.74
BDR		C5	PHE	157	CZ	4.78
BDR		C3	PHE	157	CZ	3.42
BDR		C2	PHE	157	CZ	3.88
BDR		C1	PHE	157	CZ	4.74
BDR		C4	PHE	157	CZ	4.56
BDR		C5	PHE	157	CE2	4.10
BDR		C3	PHE	157	CE2	3.39
BDR		C2	PHE	157	CE2	3.97
BDR		C1	PHE	157	CE2	4.40
BDR		C4	PHE	157	CE2	4.10
CYT		C6	PHE	157	CE2	4.36
BDR		C3	PHE	157	CE1	4.21
BDR		C2	PHE	157	CE1	4.18
BDR		C5	PHE	157	CD2	4.79
BDR		C3	PHE	157	CD2	4.15
BDR		C2	PHE	157	CD2	4.35
BDR		C1	PHE	157	CD2	4.40
BDR		C4	PHE	157	CD2	4.66
CYT		C6	PHE	157	CD2	3.56
CYT		C5	PHE	157	CD2	3.94
CYT		C4	PHE	157	CD2	4.53
CYT		C2	PHE	157	CD2	4.55
BDR		C2	PHE	157	CD1	4.52
CYT		C6	PHE	157	CD1	4.57
CYT		C5	PHE	157	CD1	4.65
CYT		C4	PHE	157	CD1	4.53
CYT		C2	PHE	157	CD1	4.30
BDR		C2	PHE	157	CG	4.63
BDR		C1	PHE	157	CG	4.73
CYT		C6	PHE	157	CG	3.68
CYT		C5	PHE	157	CG	3.69
CYT		C4	PHE	157	CG	3.87
CYT		C2	PHE	157	CG	4.13
CYT		C6	PHE	157	CB	3.59
CYT		C5	PHE	157	CB	3.05
CYT		C4	PHE	157	CB	3.09
CYT		C2	PHE	157	CB	4.22
CYT		C5	PHE	157	C	4.47
CYT		C4	PHE	157	C	4.10
CYT		C5	PHE	157	CA	4.35
CYT		C4	PHE	157	CA	4.22
BDR		C3	ASP	54	CG	4.48
PHO		C5	ASP	54	CG	4.50
BDR		C5	ASP	54	CB	4.55
BDR		C3	ASP	54	CB	4.53
PHO		C5	ASP	54	CB	4.11

21		Atom1	Residues		Atom2	Distance
BDR		C3	SER	251	CB	4.39
BDR		C4	SER	251	CB	4.73
PHO		C5	SER	251	CB	3.99
BDR		C3	SER	251	CA	4.67
BDR		C4	SER	251	CA	4.65
PHO		C5	SER	251	CA	4.19
BDR		C5	ASN	250	CB	4.08
BDR		C4	ASN	250	CB	4.37
PHO		C5	ASN	250	CB	3.78
BDR		C5	ASN	250	C	4.78
BDR		C4	ASN	250	C	4.67
PHO		C5	ASN	250	C	4.06
PHO		C5	ASN	250	CA	4.07
PHO		C5	THR	249	C	4.78
BDR		C2	LYS	215	CE	4.58
BDR		C2	TYR	210	CZ	4.13
BDR		C1	TYR	210	CZ	3.56
ADE		1C21	TYR	210	CZ	4.49
ADE		C2	TYR	210	CZ	4.03
BDR		C2	TYR	210	CE2	4.22
BDR		C1	TYR	210	CE2	4.15
BDR		C1	TYR	210	CE1	4.48
ADE		1C21	TYR	210	CE1	3.83
ADE		C2	TYR	210	CE1	4.11
ADE		1C21	TYR	210	CD1	4.69
ADE		C6	HIS	209	CE1	3.81
ADE		C4	HIS	209	CE1	3.11
ADE		C2	HIS	209	CE1	4.09
ADE		C41	HIS	209	CE1	3.31
BDR		C2	HIS	209	CD2	4.45
BDR		C1	HIS	209	CD2	4.67
ADE		C6	HIS	209	CD2	3.28
ADE		C4	HIS	209	CD2	3.60
ADE		C2	HIS	209	CD2	3.87
ADE		C41	HIS	209	CD2	4.43
ADE		1C21	HIS	209	CG	4.50
ADE		C6	HIS	209	CG	3.81
ADE		C4	HIS	209	CG	3.47
ADE		C2	HIS	209	CG	3.63
ADE		C41	HIS	209	CG	3.99
ADE		1C21	HIS	209	CB	4.56
ADE		C6	HIS	209	CB	4.68
ADE		C4	HIS	209	CB	4.32
ADE		C2	HIS	209	CB	4.02
ADE		C41	HIS	209	CB	4.77
ADE		1C21	ASP	206	CG	3.97
ADE		1C21	ASP	206	CB	4.53
ADE		1C21	ASP	206	CA	3.97
ADE		1C21	VAL	205	CG1	4.32
ADE		C4	VAL	205	CG1	4.52
ADE		C41	VAL	205	CG1	3.25
ADE		C41	VAL	205	CB	4.61
ADE		1C21	VAL	205	C	4.60
ADE		C41	VAL	205	C	4.77
BDR		C5	PHE	157	CZ	4.66
BDR		C3	PHE	157	CZ	4.18
BDR		C2	PHE	157	CZ	4.58
BDR		C1	PHE	157	CZ	4.76
BDR		C4	PHE	157	CZ	4.71
ADE		C6	PHE	157	CZ	3.20
BDR		C5	PHE	157	CE2	4.24
BDR		C3	PHE	157	CE2	4.47
BDR		C1	PHE	157	CE2	4.79
BDR		C4	PHE	157	CE2	4.55
ADE		C6	PHE	157	CE2	3.42
ADE		C6	PHE	157	CE1	3.00
ADE		C4	PHE	157	CE1	4.42
ADE		C6	PHE	157	CD2	3.44
ADE		C4	PHE	157	CD2	4.30
ADE		C6	PHE	157	CD1	3.00
ADE		C4	PHE	157	CD1	3.77
ADE		C2	PHE	157	CD1	4.73
ADE		C41	PHE	157	CD1	4.63
ADE		C6	PHE	157	CG	3.26
ADE		C4	PHE	157	CG	3.71
ADE		C2	PHE	157	CG	4.65
ADE		C41	PHE	157	CG	4.42
ADE		C6	PHE	157	CB	4.13
ADE		C4	PHE	157	CB	3.79
ADE		C41	PHE	157	CB	3.99
BDR		C5	ASP	54	CG	4.35
BDR		C3	ASP	54	CG	4.00
PHO		C5	ASP	54	CG	4.22
BDR		C5	ASP	54	CB	4.03
BDR		C3	ASP	54	CB	4.49
PHO		C5	ASP	54	CB	4.01

23		Atom1	Residues		Atom2	Distance
PHO		C5	SER	251	CB	4.53
PHO		C5	SER	251	CA	4.64
PHO		C5	ASN	250	CG	4.74
BDR		C5	ASN	250	CB	4.28
BDR		C4	ASN	250	CB	4.56
PHO		C5	ASN	250	CB	3.70
PHO		C5	ASN	250	C	4.31
PHO		C5	ASN	250	CA	4.21
BDR		C2	TYR	210	CZ	4.20
BDR		C1	TYR	210	CZ	4.06
BDR		C2	TYR	210	CE2	4.64
BDR		C1	TYR	210	CE1	4.71
HYP		C8	HIS	209	CE1	3.36
HYP		C5	HIS	209	CE1	4.46
HYP		C8	HIS	209	CD2	3.54
BDR		C2	HIS	209	CD2	4.10
HYP		C8	HIS	209	CG	3.41
BDR		C2	HIS	209	CG	4.56
HYP		C8	HIS	209	CB	4.14
HYP		C5	VAL	205	CG1	4.69
HYP		C6	ASN	158	CG	3.96
HYP		C6	ASN	158	CA	4.62
HYP		C4	PHE	157	CZ	4.64
BDR		C5	PHE	157	CZ	4.47
BDR		C3	PHE	157	CZ	3.54
BDR		C2	PHE	157	CZ	4.00
BDR		C1	PHE	157	CZ	4.52
BDR		C4	PHE	157	CZ	4.39
HYP		C2	PHE	157	CE2	4.73
HYP		C4	PHE	157	CE2	4.00
BDR		C5	PHE	157	CE2	3.86
BDR		C3	PHE	157	CE2	3.68
BDR		C2	PHE	157	CE2	4.24
BDR		C1	PHE	157	CE2	4.31
BDR		C4	PHE	157	CE2	4.04
HYP		C4	PHE	157	CE1	4.66
HYP		C8	PHE	157	CE1	4.50
BDR		C3	PHE	157	CE1	4.31
BDR		C2	PHE	157	CE1	4.31
HYP		C2	PHE	157	CD2	3.71
HYP		C4	PHE	157	CD2	3.28
HYP		C8	PHE	157	CD2	4.40
HYP		C5	PHE	157	CD2	3.88
HYP		C6	PHE	157	CD2	4.39
BDR		C5	PHE	157	CD2	4.67
BDR		C3	PHE	157	CD2	4.54
BDR		C2	PHE	157	CD2	4.74
BDR		C1	PHE	157	CD2	4.44
BDR		C4	PHE	157	CD2	4.70
HYP		C4	PHE	157	CD1	4.05
HYP		C8	PHE	157	CD1	3.98
HYP		C5	PHE	157	CD1	4.08
BDR		C2	PHE	157	CD1	4.79
HYP		C2	PHE	157	CG	4.06
HYP		C4	PHE	157	CG	3.31
HYP		C8	PHE	157	CG	3.94
HYP		C5	PHE	157	CG	3.40
HYP		C6	PHE	157	CG	3.90
BDR		C1	PHE	157	CG	4.76
HYP		C2	PHE	157	CB	3.67
HYP		C4	PHE	157	CB	3.39
HYP		C8	PHE	157	CB	4.14
HYP		C5	PHE	157	CB	2.95
HYP		C6	PHE	157	CB	2.89
HYP		C5	PHE	157	C	4.49
HYP		C6	PHE	157	C	3.77
HYP		C5	PHE	157	CA	4.30
HYP		C6	PHE	157	CA	3.94
HYP		C2	THR	155	CG2	3.95
HYP		C2	THR	155	CB	4.02
BDR		C5	ASP	54	CG	4.64
BDR		C3	ASP	54	CG	4.21
PHO		C5	ASP	54	CG	4.38
BDR		C5	ASP	54	CB	4.14
BDR		C3	ASP	54	CB	4.44
PHO		C5	ASP	54	CB	4.03
